# Canagliflozin retards age-related lesions in heart, kidney, liver, and adrenal gland in genetically heterogenous male mice

**DOI:** 10.1007/s11357-022-00641-0

**Published:** 2022-08-16

**Authors:** Jessica M. Snyder, Kerriann M. Casey, Andrzej Galecki, David E. Harrison, Hashan Jayarathne, Navasuja Kumar, Francesca Macchiarini, Nadia Rosenthal, Marianna Sadagurski, Adam B. Salmon, Randy Strong, Richard A. Miller, Warren Ladiges

**Affiliations:** 1 Department of Comparative Medicine, School of Medicine, University of Washington, Seattle, WA USA; 2 University of Washington Health Sciences Center, I-446 Box 357350, WA 98195 Seattle, USA; 3 Department of Comparative Medicine, Stanford University, CA Stanford, USA; 4 Department of Internal Medicine, Medical School, University of Michigan, Ann Arbor, MI USA; 5 The Jackson Laboratory, Bar Harbor, ME USA; 6 Department of Biological Sciences, Wayne State University, MI Detroit, USA; 7 Geriatrics Center, University of Michigan, Ann Arbor, MI USA; 8 Division of Aging Biology, National Institute On Aging, Bethesda, MD USA; 9 National Heart and Lung Institute, Imperial College, London, UK; 10 Geriatric Research, Education and Clinical Center and Research Service, South Texas Veterans Health Care System, San Antonio, TX USA; 11 Barshop Institute for Longevity and Aging Studies and Department of Molecular Medicine, The University of Texas Health Science Center, San Antonio, TX USA; 12 Barshop Institute for Longevity and Aging Studies and Department of Pharmacology, The University of Texas Health Science Center, San Antonio, TX USA; 13 Pathology and Geriatrics Center, University of Michigan, Ann Arbor, MI USA

**Keywords:** Age-related pathology, Canagliflozin, Lifespan, ITP, Sexual dimorphism

## Abstract

Canagliflozin (Cana), a clinically important anti-diabetes drug, leads to a 14% increase in median lifespan and a 9% increase in the 90th percentile age when given to genetically heterogeneous male mice from 7 months of age, but does not increase lifespan in female mice. A histopathological study was conducted on 22-month-old mice to see if Cana retarded diverse forms of age-dependent pathology. This agent was found to diminish incidence or severity, in male mice only, of cardiomyopathy, glomerulonephropathy, arteriosclerosis, hepatic microvesicular cytoplasmic vacuolation (lipidosis), and adrenal cortical neoplasms. Protection against atrophy of the exocrine pancreas was seen in both males and females. Thus, the extension of lifespan in Cana-treated male mice, which is likely to reflect host- or tumor-mediated delay in lethal neoplasms, is accompanied by parallel retardation of lesions, in multiple tissues, that seldom if ever lead to death in these mice. Canagliflozin thus can be considered a drug that acts to slow the aging process and should be evaluated for potential protective effects against many other late-life conditions.

**Supplementary Information:**

The online version contains supplementary material available at 10.1007/s11357-022-00641-0.

## Introduction

Canagliflozin (Cana), an inhibitor of sodium glucose cotransporter 2 (SGLT2), is used clinically as an oral hypoglycemic agent in the treatment of type 2 diabetes. Recently, this drug has been shown to extend lifespan in genetically heterogeneous male mice, but to provide no lifespan benefit to females at the dose used [1]. Cana has also been shown to prevent carcinogenesis in mouse models of diabetes and non-alcoholic steatohepatitis-related hepatocarcinogenesis and pancreatic cancer [2–4]. Cana is renoprotective in mouse models of type 2 diabetes and cisplatin renotoxicity, and cardioprotective in mouse models of diabetes [5–7]. Additionally, Cana alleviates LPS-induced lung injury, has an anti-inflammatory effect in vivo and in vitro, and extends lifespan in male rats [8–10].

The basis for the lifespan benefit seen in male mice is not known, and it is not known whether the lack of lifespan effect in females reflects diminished beneficial effects in females, or harmful side effects, in females, that oppose any potential positive effects. In a recent study, for example, Cana was found to cause a modest increase in adenoma burden in female APC-Min mice [11].

It seems plausible that the beneficial effects of Cana are related to its ability to minimize peaks in plasma glucose after consumption of meals high in carbohydrates. Cana does this by blocking renal re-absorption of glucose in conditions of hyperglycemia. Mouse lifespan is also extended significantly by acarbose, which acts to limit post-prandial glucose spikes by delaying the digestion of carbohydrates to absorbable sugar in the gastrointestinal tract. Acarbose leads to significantly longer lifespan in both males and females, but the degree of lifespan increase is much higher in males at each of three tested doses [12, 13]. UM-HET3 mice, like most laboratory mouse stocks, do not die from diabetes or its complications, nor do they show metabolic dysfunction with age, and neoplastic disease of various cell types is responsible for deaths of about 80% of UM-HET3 mice, implying that Cana, and acarbose, diminish incidence, progression, or lethality of multiple forms of neoplasm through unknown mechanisms [14, 15]. A goal of the current study is to determine whether Cana lowers the incidence or severity of age-dependent non-neoplastic diseases that do not lead to death, as a test of the idea that Cana slows aging in mice. A previous study addressed this issue in rapamycin-treated mice.

Rapamycin, an inhibitor of the kinase mTOR (“mechanistic target of rapamycin”), increases median lifespan by up to 23% in males and 26% in females [16]. Wilkinson et al., in a cross-sectional study of 22-month-old mice, showed rapamycin-dependent declines in many forms of age-related pathology, including liver degeneration, adrenal neoplasms, age-related changes in cardiomyocytes, and loss of tendon elasticity [16]. Application of a validated protocol for assessment of age-sensitive lesions can provide a comprehensive picture of the extent to which an intervention that extends lifespan also retards pathological change in many distinct tissues and organs [17].

The purpose of this study was therefore to analyze cross-sectional pathology in a cohort of female and male mice treated with Cana and sacrificed at 22 months of age. Specifically, the goal of the study was to see if there was evidence that Cana slows aspects of aging that are degenerative rather than neoplastic in nature (cardiomyopathy, glomerulopathy) and also whether Cana reduced the presence of neoplasia in a variety of organs in 22-month-old mice.

## Methods

### Mouse husbandry

The Intervention Testing Program (ITP) protocol for longevity studies has been previously described [18, 19]. In brief, mice are bred as the progeny of (BALB/cByJ × C57BL/6 J)F1 mothers (JAX #100,009) and (C3H/HeJ × DBA/2 J)F1 fathers (JAX #100,004), so that each mouse is genetically unique and a full sibling to all other mice with respect to segregating nuclear alleles. Mice are housed at three males or four females per cage from weaning and are provided food (Purina 5LG6) and water ad libitum. Cana was administered in the feed at 180 ppm beginning at 7 months of age until sacrifice at 22 months of age. Mice were weighed every 6 months but were otherwise undisturbed. Sentinel mice were tested for antibodies against specified viruses and for parasites, either annually or quarterly depending on the pathogen, and all such tests were negative at each test site throughout the period of this study.

### Pathology

At 22 months of age, mice were euthanized using carbon dioxide asphyxiation. Following euthanasia, incisions were made in the cranium, thorax, and abdomen, and the specimens were immersed in 10% neutral-buffered formalin for storage at room temperature. Random samples from each of the three sites were then sent to the University of Washington for gross inspection and for preparation of slides for histological examination. During gross examination and dissection, all obvious external and internal abnormalities were recorded. Two hundred twenty-eight mice from three separate colonies were examined histologically: TJL (40 male [20 Cana; 20 control]; 40 female [21 Cana; 19 control]); UM (34 male [24 Cana; 10 control]; 42 female [20 Cana; 22 control]); and UT (40 male [19 Cana; 21 control]; 32 female [18 Cana; 14 control]). Tissues evaluated by histologic examination included brain (three coronal sections at the level of the striatum; hippocampus/thalamus; and cerebellum); lung; heart; kidney; adrenal glands; liver; spleen; pancreas; mesenteric fat and lymph node(s); thyroid gland; reproductive tract (consisting of uterus and ovaries for female mice and testis, epididymis and seminal vesicles for male mice); three longitudinal sections of skin at the level of the interscapular region, dorsal mid thoracic region, dorsal lumbar region, and pinna; and any additional lesions noted grossly. Tissues were routinely processed, and paraffin embedded, after which 4–5 micron sections were stained with hematoxylin and eosin (H/E) for histological assessment. Heart, kidney, and liver slides from some mice were also stained with Masson’s trichrome.

Some lesions were recorded as present (1) or absent (0): all neoplasms; thalamic mineralization; thalamic intracytoplasmic inclusions; respiratory epithelial hyperplasia; mineralization of vessels within the lung; foci of cellular alteration within the liver; hepatocellular hypertrophy/hyperplasia; pancreatic islet cell hyperplasia; bladder dilation; ovarian cyst (a score of 2 was possible if both ovaries were cystic); ovarian hyperplasia/neoplasia; adrenal cortical hyperplasia; and thyroid follicular hyperplasia. Other lesions were graded on a 0 (absent) to 4 (severe) scale, with a score of 1 indicating minimal; 2 indicating mild; and 3 and 4 indicating moderate and severe lesions, respectively. These lesions included lung lymphoid aggregates; cardiomyopathy; arteriosclerosis of cardiac vessels; glomerulonephropathy; lymphoid aggregates within the kidney; macrovesicular cytoplasmic vacuolation of the hepatocytes (lipidosis, presumptive); microvesicular cytoplasmic vacuolation of the hepatocytes (lipidosis, presumptive); hepatic necrosis/hepatocellular degeneration; hepatic angiectasis/telangiectasia; hepatocellular inflammatory cell infiltration; microgranulomas within the liver; lymphoid aggregates within the liver; pancreatic exocrine atrophy; pancreatic inflammation; pancreatic lymphoid accumulations; adrenal pigment, atrophy, and degeneration; subcapsular spindle cell hyperplasia (adrenal); ovarian atrophy, lipofuscin, and degeneration; ovarian angiectasia; testicular atrophy/degeneration; uterine cystic endometrial hyperplasia; uterine angiectasia; uterine inflammation; and “cold follicles” of the thyroid (defined as dilated colloid-filled follicles with flattened epithelium). For paired organs, the more severe organ score was recorded. Autolysis in some organs (most commonly kidney, pancreas, and spleen) from some mice precluded scoring, and scoring also was not performed if the organ was not present or only a small section of the organ was obtained (most commonly adrenal gland and thyroids). Scoring was performed as previously described [1] including criteria previously developed and validated to assess lesions of the kidney, heart, liver, and lung in aged mice [17]. Detailed scoring criteria for cardiomyopathy, microvesicular cytoplasmic vacuolation of the hepatocytes, glomerulonephropathy, and arteriosclerosis of cardiac vessels is presented in Supplemental Table 1.

Adrenal cortical neoplasms in this study were diagnosed on the basis of the degree of compression of the adjacent adrenal parenchyma, disruption of the normal cortical architecture, and/or extension above the normal contour of the adrenal surface. A similar percentage of adrenal tumors were observed in this study compared to previous studies [16].

An initial analysis of all slides was conducted by a board-certified veterinary pathologist (JMS), blinded to treatment group. Because this process involved necropsies and histologic assessment of 7–8 slides for each of 228 mice and took place over an extended interval (6 months), the original pathologist did a second blinded grading of male heart and kidney slides for the following lesions: cardiomyopathy, arteriosclerosis, and glomerulopathy. As an additional check for consistency, a second board certified veterinary pathologist (KMC), also blinded to treatment groups, independently scored cardiomyopathy, arteriosclerosis, glomerulonephropathy, and adrenal cortical neoplasms in the slides from the male mice. With some variations, all three sets of scores were similar, and all were consistent with the key conclusion that Cana delayed age-dependent pathology in males. The original scores of the initial pathologist were used for all statistical analyses presented in results.

### Statistics

To test for association between treatment and lesion scores, we calculated the chi-squared statistics for the 2 × *N* table for each lesion, separately for each sex, pooling across the three test sites. For lesions scored as present or absent, this was a 2 × 2 table; for lesions graded on a scale from 0 to 2, or 4, the corresponding 2 × *N* table was calculated instead. Reported *P* values are not corrected for multiple comparisons. In some cases, 25% of more of the table cells had fewer than 5 expected cases, and in these instances, the Fisher’s exact test was used instead, because the chi-squared test may be invalid. Statistical analyses were conducted using SAS 9.4 (SAS Institute, Cary, NC). The water consumption data were analyzed using a two-factor ANOVA (sex, drug, and interaction).

## Results

**Fig. 1:**
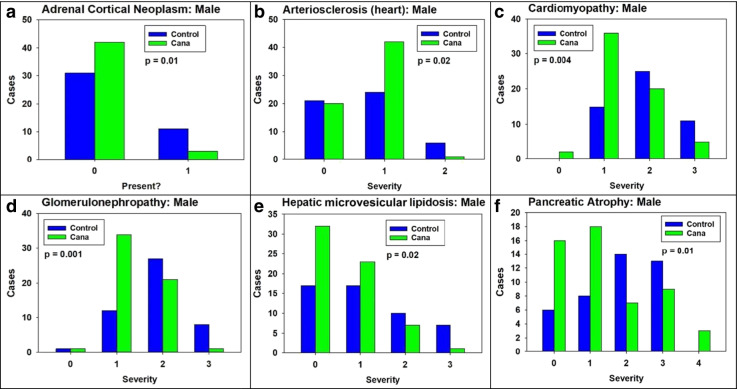
The incidence or severity of age-related lesions was decreased in 22-month-old HET3 male mice treated with canagliflozin starting at 7 months of age. **A** Adrenal gland – cortical neoplasm, **B** Heart – arteriosclerosis, **C** Heart – cardiomyopathy, **D** Kidney – glomerulonephropathy, **E** Liver – microvesicular cytoplasmic vacuolation (lipidosis), and **F** Pancreas – exocrine atrophy

Mice were scored for severity of 38 lesions in males and 46 lesions in females by a board-certified veterinary pathologist, blind to treatment condition, as detailed in “Methods” section. Some lesions were scored as present or absent (score 0–1), while others were graded on scales from 0 to 2, or 0 to 4. Supplemental Table 2 presents the lesions scored in each sex, with a tabulation of the number of non-zero scores for each lesion. Because these scores are ordinal, rather than interval variables, we used the chi-squared test to evaluate differences between control and Cana mice for each lesion, except that the Fisher’s exact test was used when 25% or more of the cells had 5 or fewer cases. Cana significantly diminished the incidence or severity of graded lesions in six lesions representing five organs in male mice, as shown by the distribution of lesion scores in Fig. 1.

**Fig. 2:**
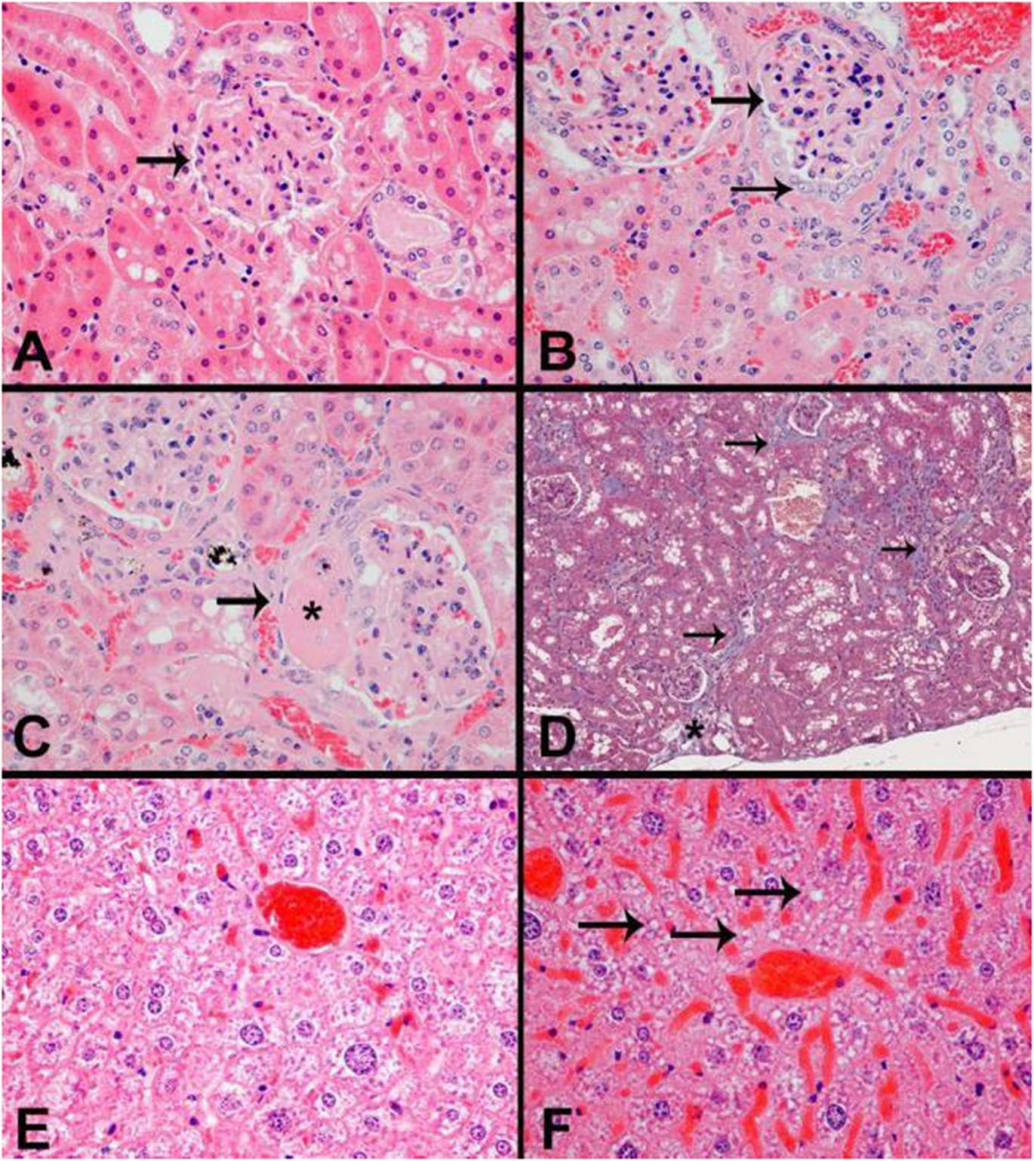
Histology of the kidney and liver. Kidney (**A**–**D)**. Representative histology images consistent with minimal glomerulonephropathy (**A**, grade 1); mild glomerulonephropathy (**B**, grade 2); and moderate glomerulonephropathy (**C**, grade 3) with increased mesangial matrix (asterisk) are shown. HE, original magnification 40 × . Arrows indicate the glomerulus. **D** Masson’s trichrome staining of a kidney with moderate glomerulonephropathy shows an irregular and focally depressed capsular surface (asterisk) and increased collagen deposition (blue staining, arrows) within the interstitium. Original magnification 10 ×. Liver (**E-F)**. Microvesicular cytoplasmic vacuolation in male mice. **E** Normal liver without cytoplasmic vacuolation is shown (histologic grade 0). **F** Minimal centrilobular cytoplasmic microvesicular vacuolation (histologic grade 1)

**Fig. 3:**
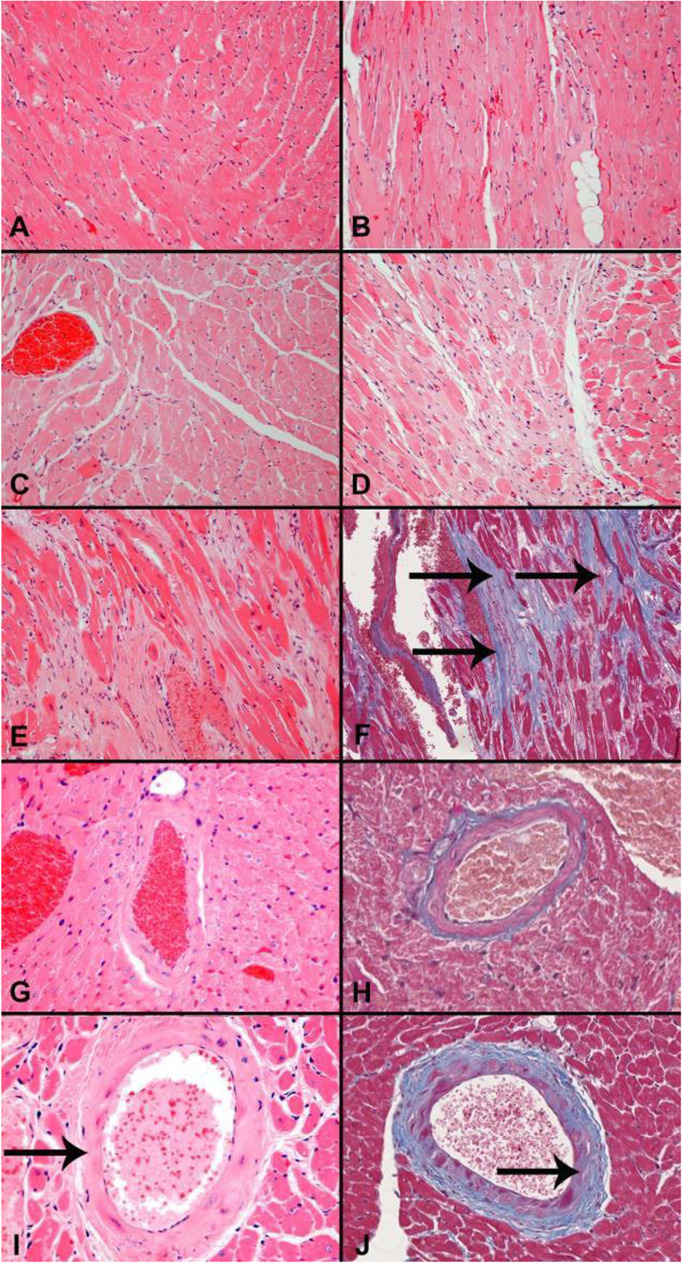
Histology of the heart and myocardial vessels. Heart (**A**–**E**) (HE, original magnification 20 ×) and **F** (Masson’s trichrome, original magnification 10 ×). **A** Normal myocardium (grade 0). **B** Minimal cardiomyopathy (grade 1). **C** Mild cardiomyopathy (grade 2). **D** Moderate cardiomyopathy (grade 3). **E** Severe cardiomyopathy with Masson’s trichrome (**F**) demonstrating regions of increased collagen deposition (blue staining, arrows) within the myocardium. **G**–**J** Myocardial vessels. **G**–**H** HE (**G** and **I**, original magnification 40 ×) and Masson’s trichrome (**H** and **J**, original magnification 20 ×). Normal myocardial vessels (arteriosclerosis grade 0) stained with HE (**G**) and Masson’s trichrome (**H**). **I**–**J** Arteriosclerosis (grade 2) stained with HE (**I**) and Masson’s trichrome (**J**)

**Fig. 4:**
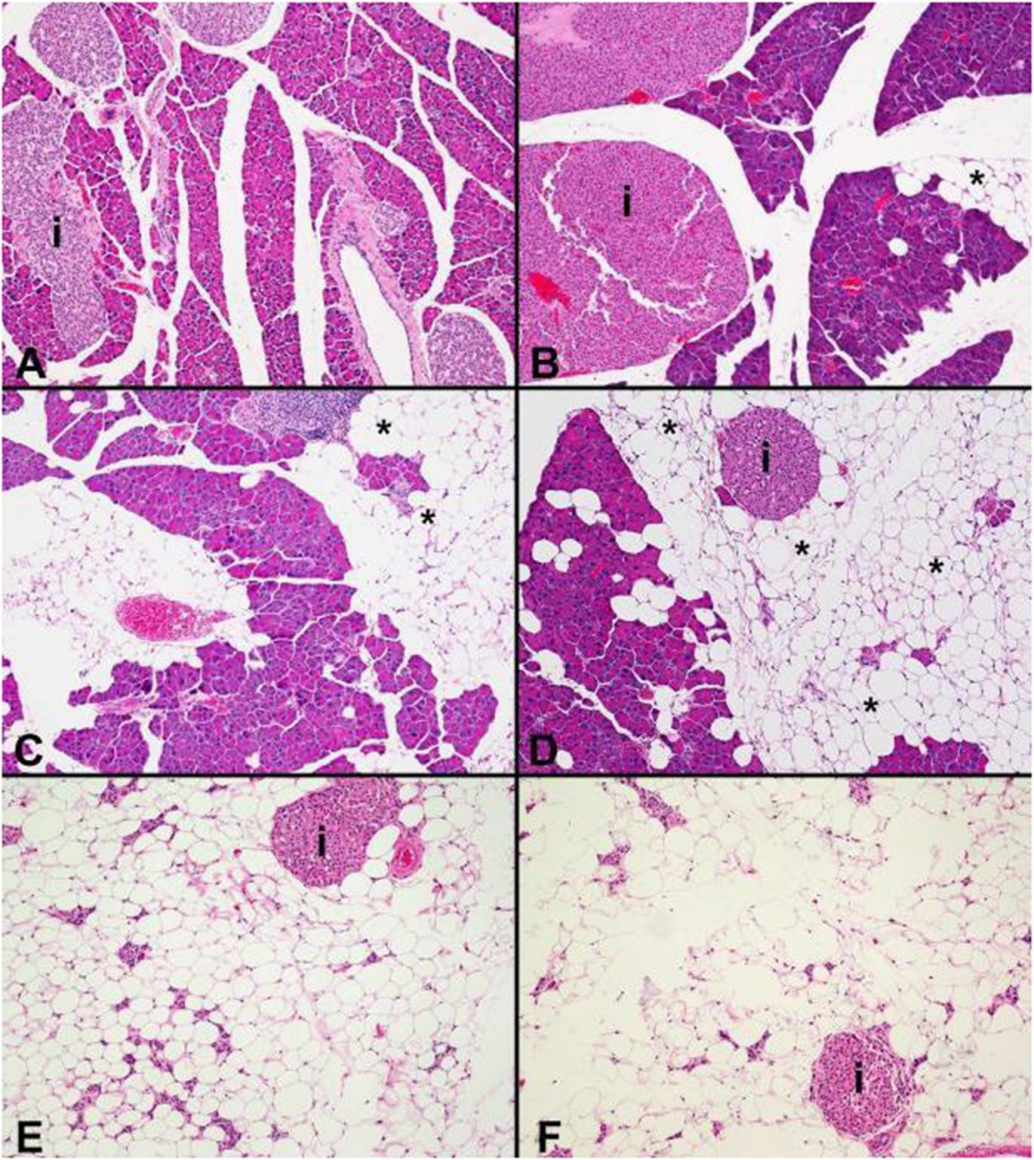
Histology of the pancreas demonstrating increasing severity of exocrine pancreatic atrophy and replacement with fat (asterisks). HE, original magnification 10 × . Islets indicated with “*i.*” **A** Normal pancreas (grade 0). **B** Grade 1, with minimal focal exocrine atrophy and replacement with adipose tissue (asterisk). Islet cell hyperplasia is also present. **C** Grade 2, mild exocrine atrophy. **D** Grade 3, moderate exocrine atrophy. **E**, **F** Grade 4, severe exocrine atrophy with regionally extensive near total loss of exocrine pancreatic tissue. Islets (*i*) and pancreatic ducts remain

Histological images representative of lesion severity grades for kidney and liver are shown in Fig. 2. Histologic changes in the kidney were consistent with an age-related glomerulonephropathy, characterized by increased expansion of the glomerulus mesangial matrix, interstitial inflammatory cell infiltration and/or fibrosis, and tubular changes including increased intraluminal protein, which were more severe in control (median histologic score of 2, Fig. 2B) compared to Cana-treated (median histologic score of 1, Fig. 2A) male mice. Microvesicular cytoplasmic vacuolation, consistent with lipidosis, was also increased in the livers of control (median histologic score of 1, Fig. 2F) compared to Cana-treated (median histologic score of 0, Fig. 2E) male mice. Histological images representative of lesion severity grades for the cardiovascular system are shown in Fig. 3. Cardiac changes were characterized by cardiomyocyte degeneration and fibrosis, which were less severe in Cana-treated male mice (median score 1, Fig. 3B) compared to controls (median score 2, Fig. 3C). Examples of the severity grades for pancreas exocrine atrophy, which was also less severe in Cana-treated (median histologic score 1, Fig. 4B) versus control male mice (median histologic score 2, Fig. 4C), are shown in Fig. 4.

Cana thus suppresses either the incidence or severity of mid-life pathology in adrenal gland, cardiac vessels, myocardium, kidney, liver, and pancreas in HET3 male mice. It is noteworthy that these forms of pathology do not, except in very rare cases, progress to the point that they cause death of the mice [20], most of which die of malignant neoplasms, most commonly hematopoietic neoplasms (lymphoma, histiocytic sarcoma); brochiolo-alveolar carcinomas; hepatocellular carcinomas; and hemangiosarcomas. These data thus indicate that Cana diminishes age-related degenerative changes in multiple tissues of male mice.

**Fig. 5:**
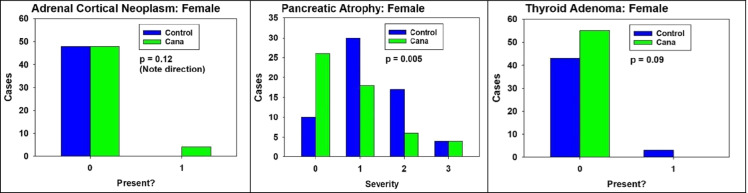
Canagliflozin influenced histologic lesions in female mice. **A** Adrenal gland – cortical neoplasm. **B** Pancreas – exocrine atrophy. **C** Thyroid – adenoma

In females, there were three lesions where Cana-treated mice differed from controls to a degree that approached or achieved statistical significance. These are shown in Fig. 5. Adrenal cortical neoplasia was seen in 4/52 Cana females, but not seen in any of 48 control females. By the Fisher’s exact test, *p* = 0.12. This is suggestive, though not definitive, evidence that Cana may increase adrenal cortical neoplasms in female mice, in contrast to the lower incidence of these neoplasms in Cana-treated male mice (Fig. 1). Cana diminishes the severity of pancreatic exocrine atrophy in females (*p* = 0.005), just as it does in male mice (Fig. 1). Thyroid adenoma is seen in 3/46 control females, and in none of 55 Cana females, a difference which is suggestive for a protective effect, but does not reach statistical significance (*p* = 0.09 by Fisher’s exact test). Thus, with the exception of pancreatic exocrine atrophy, the multi-organ protective effect produced by Cana in male mice is not seen in females.

**Fig. 6:**
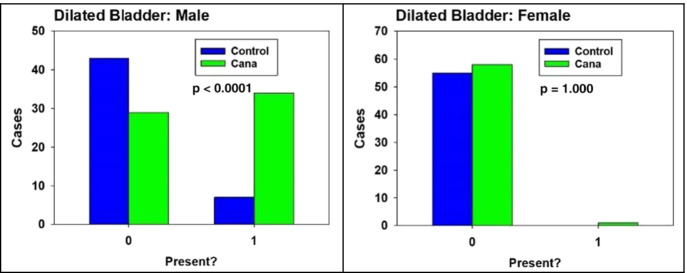
Canagliflozin affects bladder size in male but not female mice

Observation of mice receiving Cana showed greatly increased drinking and urination compared to control mice. This observation was noted in both sexes at all three sites. Table 1 shows values for water consumption over a 24-h period for each group, measured in 24-month-old animals (5 per sex for Cana and control). Two-factor ANOVA showed significant effects for sex (*p* = 0.007, females drink more) and for drug treatment (*p* = 0.0009, Cana drink more than controls). The interaction term is not significant, so there is no evidence that the effect of Cana on water intake is different between the sexes. Necropsy data showed a much greater incidence of bladder dilation in male mice; this finding was noted in 34/63 Cana males but only 7/50 control males (*p* < 0.0001) (Fig. 6). It seems possible that this lesion results from the greatly increased daily urine volume in these mice. Curiously, dilated bladder is seen much less often in female mice: 1/59 in Cana females and 0/55 in control females. The reason for this sex-specific Cana-associated increase is uncertain but may result from anatomic factors relating to the urinary sphincter and/or urethra, or other factors. This lesion did not appear histologically to be significantly associated with urinary outflow obstruction causing hydronephrosis/hydroureter or inflammation of the bladder or kidneys. Cystitis was noted in 2 control and none of the Cana-treated male mice. Hydronephrosis was noted histologically in 3 Cana-treated male mice and 1 control male mouse, and pyelonephritis/pyelitis was observed in 1 control and 2 Cana-treated male mice.

**Table 1:** Mean water intake for 24-h period in 24-month-old mice. Values are mean ± SEM for *N* = 5, in milliliters per 24 h

	Males	Females
Control	1.7 ± 0.7	2.9 ± 0.5
Cana	3.6 ± 1.0	7.4 ± 0.9

To confirm the results of male-related pathology in Cana-treated mice, changes in the heart (cardiomyopathy, arteriosclerosis), kidney (glomerulonephropathy), and adrenal (cortical neoplasia) were re-scored blindly by the original pathologist, and also scored blindly by a second pathologist. Results of the three evaluations were similar for all parameters except for arteriosclerosis, which did not differ significantly between control and Cana-treated male mice on rescoring performed by the original pathologist and the second pathologist and for which there was less agreement between pathologists on scores (Supplemental Fig. 1).

## Discussion

The central goal of this study was to test the idea that canagliflozin would delay age-dependent pathology in multiple organs and would do so in a sex-specific fashion. Since most laboratory stocks of mice, including the UM-HET3 mice used by the ITP, die of neoplastic disease, then lifespan extension in drug-treated mice could, in principle, reflect a specifically anti-neoplastic protective effect, due to drug effects on tumor cells, host defenses, or both [15]. Evidence that a drug, diet, or mutant gene slows the entire aging process is based on evidence for protective effects on a wide range of age-sensitive traits, among which data on pathological lesions can be particularly persuasive. Calorie-restricted diets, Snell and Ames pituitary mutations, and more recently rapamycin have all met such a challenge, with delay or deceleration in multiple age-sensitive endpoints [16, 21, 22]. In these earlier situations, the lifespan extension and opposition to age effects were equally apparent in both sexes. In contrast, the effect of Cana on lifespan of UM-HET3 mice is seen exclusively in male mice [1]. Although the basis for the sex specificity of Cana on lifespan is not known, this model provides an opportunity to see if age-sensitive traits, including multiple forms of pathology, are also altered in a sex-specific way at a cross-sectional time point of 22 months. Our main findings were consistent with the idea that Cana retards development of lesions principally in males, with sex-specific reductions in lesion scores in heart muscle, adrenal glands, cardiac vessels, liver, and kidney. Cana diminished atrophy of the exocrine pancreas equally in both sexes, the only lesion for which beneficial effects were seen in females as well as males. The male-specific lifespan effect almost certainly reflects delay or deceleration of multiple forms of lethal neoplastic disease, the principal causes of death in UM-HET3 mice. Our new data establish that the drug also delays or decelerates multiple forms of age-dependent illness in males, including non-lethal lesions, supporting the conclusion that Cana slows the aging process, in mice, in a sex-specific fashion.

Our working hypothesis is that the beneficial effects of Cana reflect its ability to diminish post-prandial peak plasma glucose concentrations, although Cana has also been shown to modify other pathways relevant to increased lifespan and healthspan such as mTOR signaling, AMPK signaling, and FGF21 levels [1]. Recently, Cana was shown to significantly improve central insulin sensitivity in the hypothalamus and the hippocampus of aged male mice and to decrease inflammatory cytokine production by microglia [23]. This is in line with findings from the ITP showing that acarbose, which lowers peak glucose by inhibition of bacterial hexokinases in the gastrointestinal tract, also leads to preferential lifespan effects in males. If this speculation is correct, then studies focused on the relationship of peak glucose levels to aging, cancer, lifespan, and other age-specific forms of pathology are likely to be revealing. It is possible, however, that some of the effects of Cana reflect inhibition of SGLT2 or SGLT1 in one or more tissues, perhaps in addition to its systemic effect on glucose excursions. It is noteworthy that acarbose leads to inhibition of mTORC1 function to an equal degree in males and in females, as well as to sex-non-specific augmentation of selective sets of mRNAs, in the liver and kidney, through cap-independent translation [24]. Similarly, acarbose inhibits two important intracellular MAPK cascades: the ERK1/2 pathway leading to phosphorylation of eIF4E, a facilitator of cap-dependent translation, and the p38 MAPK cascade leading to production of acute phase inflammatory proteins; both of these effects are seen in females as well as males [25]. It will be interesting to learn if Cana-treated mice also exhibit these changes in cell signaling pathways, and, if so, whether these effects are sex-independent in all organs. Acarbose did improve some age-dependent health-related traits, including changes in grip strength and rotarod test performance, in both sexes, and led to male-specific improvements in some others, including protein ubiquitination, cardiac hypertrophy, and indices of hypothalamic inflammation [26, 27]. How acarbose and Cana lead to a mixture of sex-specific and sex-independent cellular changes and how these lead to sex-specific protection against late-life illness and death are topics worthy of additional study.

The reductions in pathology induced by Cana are similar to those caused by rapamycin in an earlier ITP study, in which the frequency of degenerative cardiac nuclei changes, macrovesicular hepatic lipidosis, and adrenal neoplasms was lower in treated mice [16]. However, whereas rapamycin benefits female lifespan as well as male, the only significant protective effect of Cana in female mice was related to pancreatic exocrine atrophy, which may reflect Cana actions independent of pathways that modulate aging and age-related lesions in males only.

Histopathologic analysis is an important adjunct to cross-sectional and longitudinal lifespan studies. Histology can add context to aging studies and has the ability to integrate in vivo, biochemical, and molecular data [28]. Evaluation of a complete tissue set and use of a scoring system with defined criteria that has been validated helps to ensure definable, meaningful, and reproducible results [17, 29]. A geropathology grading platform for scoring age-related lesions in mice has been developed and validated [17]. The scoring performed in this study used many of the same criteria previously published. While the scoring method utilized allows for the generally efficient review and scoring of a relatively large number of lesions in a number of organs on HE-stained slides, a limitation is that quantitative analysis of parameters requiring special stains or immunohistochemistry (to evaluate amyloid deposition or apoptosis, for example) or advanced histomorphometric analysis (i.e., quantification of cardiomyocyte size) is beyond the scope of the current study. Also, when scoring large numbers of tissues over an extended period of time, diagnostic drift can occur [29]. In this study, we subjected the histopathological analysis of certain tissues to re-review both by the original pathologist and a second board-certified veterinary pathologist, and found that the protective effects of Cana on cardiomyopathy and glomerulonephropathy as well as adrenocortical neoplasms were reproducible. It would be interesting to perform additional quantitative and morphometric analyses in future studies of renoprotective and cardioprotective effects of canagliflozin.

Cardiomyopathy in rodents represents a complex of progressive changes which include degeneration of cardiomyocytes and myocardial fibrosis accompanied by degenerative changes of associated myocardial blood vessels (arteriolosclerosis) as well as valvular degeneration [30, 31]. Proposed mechanisms of cardiac aging include neurohormonal regulation (including activation of the renal-angiotensin-aldosterone system (RAAS) and decreased insulin/IGF1 signaling) and mitochondrial oxidative damage, among others [31]. In age-related glomerulopathy of mice, progressive degenerative changes of the kidney characterized by expansion of the glomerular mesangial matrix; tubular degeneration, regeneration, and proteinuria; and inflammatory cell infiltration and fibrosis of the renal interstitium are observed [32, 33]. Mechanisms of chronic renal disease also include activation of the RAAS system and oxidative damage [33–35].

In previous studies, Cana has reduced hepatic fat deposition in a mouse model of diabetes [36]. Hepatic lipidosis may be characterized by large (macrovesicular) or small (microvesicular) vesicles of fat that accumulate within the cytoplasm. In this study, there was no significant difference between macrovesicular cytoplasmic vacuolation, but there was a difference between microvesicular cytoplasmic vacuolation between Cana-treated and control mice. Mild increased microvesicular steatosis has been previously observed in aged mice and was associated with increased cholesterol and triglyceride levels in these mice [37]. In human patients, Cana reduced liver injury in non-alcoholic fatty liver disease [38].

Increased water consumption was seen in both male and female mice treated with Cana, but bladder dilation was significantly higher in male but not female Cana-treated mice. The cause for this sex difference is unknown, although it is possible that sexually dimorphic anatomic differences in the lower urinary tract and/or changes in bladder and urethral sphincter pressures may contribute. Bladder dilation in male mice has been previously observed in mice with a history of increased urine production related to diabetes mellitus (personal communication). Bladder distention has also been reported in 2 women and 7 men with polyuria due to nephrogenic diabetes insipidus [39], and it was hypothesized that bladder pressure at the end of filling may contribute to the dilatation of urinary tract and that normal detrusor contractility with large post-voiding residual volume (PVR) was a unique manifestation of this condition.

This histopathological analysis was taken to test the idea that Cana-treated mice might have delays in age-related pathology in multiple lesions, including lesions that seldom if ever prove fatal, and to see to what extent such effects might be sex-specific. The results seem clear: Cana does indeed delay age-related changes in the heart, kidney, liver, exocrine pancreas, and adrenals, and this protective effect is seen in males only, with the exception that protection against pancreatic atrophy is apparent in both sexes. Since most UM-HET3 mice die of some form of neoplastic disease, the lifespan extension produced by Cana presumably reflects postponement of multiple kinds of cancer, whether by alteration of host defenses, oncogenic processes, or both. But the current data establish that Cana, like rapamycin, calorie restriction diets, and hypopituitary mutations, can be considered as an anti-aging intervention, in that it delays many forms of lethal and non-lethal age-dependent decline. Studies of Cana effects on specific deleterious aspects of aging, such as exercise tolerance, neurodegenerative pathology, protective immunity, sensory perception, and the like, will help to define the scope and potential limits of its effects.

## Supplementary Information

Below is the link to the electronic supplementary material.

Supplementary file1 (DOCX 394 KB)

Supplementary file2 (DOCX 14.2 KB)

Supplementary file3 (DOCX 13.8 KB)
