# Supplementary material for: Canagliflozin retards age-related lesions in heart, kidney, liver, and adrenal gland in genetically heterogenous male mice
Source: GeroScience. 2022 Aug 16;45(1):385–97. doi: 10.1007/s11357-022-00641-0 (PMC9886729; doi:10.1007/s11357-022-00641-0)

Supplemental Figure 1. Score distribution for original pathology score, score by the second pathologist, and re-score by the original pathologist, for cardiomyopathy, glomerulopathy, and arteriosclerosis. These parameters were scored on a 0-4 scale. Scores that agree exactly are shaded dark green, and scores that differ by 1 point are shaded light green.


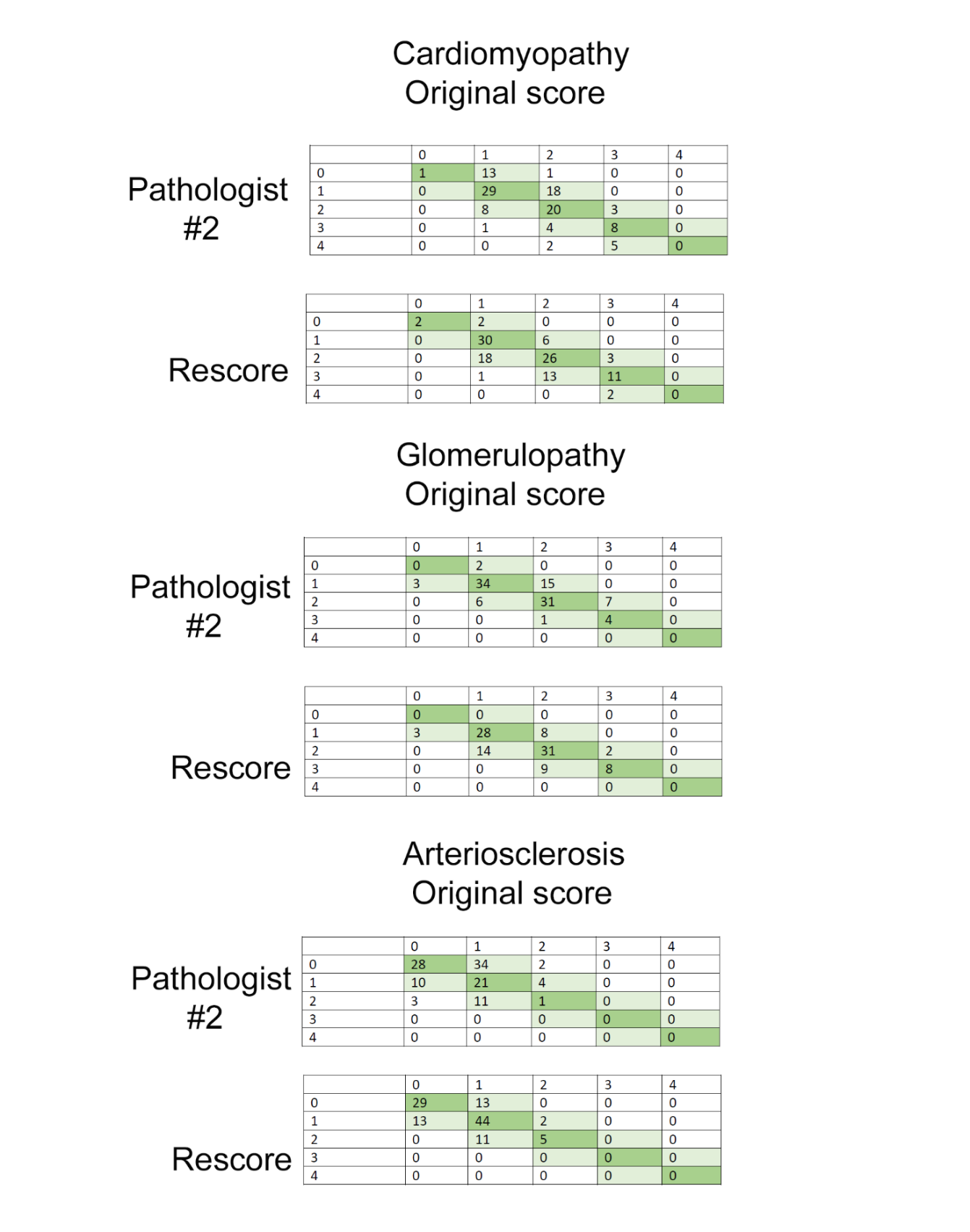

Supplement: Supplementary file 1 — Supplementary file1 (DOCX 394 KB) [file 11357_2022_641_MOESM1_ESM.docx]
