# Supplementary material for: Canagliflozin retards age-related lesions in heart, kidney, liver, and adrenal gland in genetically heterogenous male mice
Source: GeroScience. 2022 Aug 16;45(1):385–97. doi: 10.1007/s11357-022-00641-0 (PMC9886729; doi:10.1007/s11357-022-00641-0)
Supplement: Supplementary file 2 — Supplementary file2 (DOCX 14.2 KB) [file 11357_2022_641_MOESM2_ESM.docx]

Supplemental Table 1. Specific scoring criteria for arteriosclerosis, cardiomyopathy and myocardial fibrosis, glomerulonephropathy, and cytoplasmic vacuolation of the liver (lipidosis).

| Lesion | Score 1 | Score 2 | Score 3 | Score 4 |
| --- | --- | --- | --- | --- |
| Arteriosclerosis | Minimal lesions: Expansion of <10% of the vessel wall profiles [tunica media] with hyaline material  (hyaline arteriosclerosis) or laminated [“onion skin”] concentric thickening  (hyperplastic / hypertrophic arteriosclerosis) [smooth myocytes and basement membrane];  patent (non-obstructed) lumen. | Mild lesions; Expansion of <25% of the vessel wall profiles [tunica media] as above;  patent (non-obstructed) lumen. | Moderate lesions; Expansion of 25-75% of the vessel wall profiles [as described above];  partial luminal narrowing/obstruction. | Severe lesions; Expansion of >75% the vessel wall profiles as described above;  with significant luminal narrowing/obstruction; with or without mural necrosis. |
| Cardiomyopathy and myocardial fibrosis | Minimal; <5% of the myocardium affected by focal or scattered deposition of fibrous connective tissue with or without degenerative cardiomyocytes, valvular fibrosis that extends from the base of the valve into the myocardium, replacement of cardiomyocytes and mineralization. | Mild lesions; < 10% of the myocardium. | Moderate lesions; 10% to 25% of the myocardium. | Severe lesions; > 25% of the myocardium; dense deposits, replacing normal architecture. |
| Glomerulonephropathy | Less than 5% of renal parenchyma affected by segmental to global glomerular mesangial thickening with or without proteinuria, tubular degeneration/regeneration, and interstitial inflammation/fibrosis. | Mild lesions; 5-30% affected compared to grade 1 | Moderate lesions; 30 to 70% compared to grade 1. | Severe lesions; >70% compared to grade 1; glomerular obsolescence or sclerosis in addition to lesions in grade 3 |
| Cytoplasmic vacuolation of hepatocytes (lipidosis, presumptive) | Minimal; discrete microvesicular or microvesicular accumulation in hepatocytes; <5% of hepatic parenchyma involved. | Mild lesions; 5-10% affected compared to grade 1. | Moderate lesions; 10-25% compared to grade 1. | Severe lesions;  >25% affected. |
