# Supplementary material for: Canagliflozin retards age-related lesions in heart, kidney, liver, and adrenal gland in genetically heterogenous male mice
Source: GeroScience. 2022 Aug 16;45(1):385–97. doi: 10.1007/s11357-022-00641-0 (PMC9886729; doi:10.1007/s11357-022-00641-0)
Supplement: Supplementary file 3 — Supplementary file3 (DOCX 13.8 KB) [file 11357_2022_641_MOESM3_ESM.docx]

Supplemental Table 2. Number of mice in each group with lesions present from categories graded 0-1. Number of mice examined per group is listed in first row unless noted otherwise.

|  | Male control  N=51 | Male cana  N=63 | Female control  N=55 | Female cana  N=59 |
| --- | --- | --- | --- | --- |
| Thalamic mineralization | 17 (n=43) | 19 (n=58) | 12 (n=49) | 16 (n=52) |
| Thalamic intracytoplasmic inclusions | 22 (n=43) | 25 (n=58) | 23 (n=49) | 19 (n=52) |
| Bronchiolo-alveolar  adenoma | 16 | 17 | 6 | 8 |
| Bronchiolo-alveolar carcinoma | 6 | 5 | 1 | 3 |
| Bronchiolo-alveolar hyperplasia | 12 | 19 | 9 | 13 |
| Vessel mineralization, lung | 26 | 31 | 12 | 12 |
| Hepatocellular adenoma | 5 | 10 | 4 | 5 |
| Hepatocellular carcinoma | 4 | 3 | 1 | 1 |
| Focus of cellular alteration, liver | 7 | 5 | 2 | 3 |
| Hepatocellular hypertrophy/hyperplasia | 12 | 11 | 5 | 4 |
| Pancreatic islet cell hyperplasia | 32 (n=41) | 36 (n=52) | 14 (n=51) | 15 (n=54) |
| Thyroid follicular cell hyperplasia | 2 (n=46) | 8 (n=56) | 5 (n=46) | 13 (n=54) |
| Thyroid follicular cell adenoma | 0 (n=46) | 0 (n=56) | 3 (n=46) | 0 (n=54) |
| Vascular neoplasm (hemangiosarcoma) | 4 | 9 | 4 | 3 |
| Hematopoietic neoplasia | 7 | 8 | 17 | 18 |
